# Correlated lip motion and voice audio data

**DOI:** 10.1016/j.dib.2018.10.043

**Published:** 2018-10-18

**Authors:** Marco Colasito, Jeremy Straub, Pratap Kotala

**Affiliations:** a Department of Computer Science, Valdosta State University, United States; b Department of Computer Science, North Dakota State University, United States

## Abstract

This data set is comprised of correlated audio and lip movement data in multiple videos of multiple subjects reading the same text. It was collected to facilitate the development and validation of algorithms used to train and test a compound biometric system that consists of lip-motion and voice recognition. The data set is a collection of videos of volunteers reciting a fixed script that is intended to be used to train software to recognize voice and lip-motion patterns. A second video is included of the individual reciting a shorter phrase, which is designed to be used to test the recognition functionality of the system. The recordings were collected in a controlled, indoor setting with a 4K professional-grade camcorder and adjustable, LED lights.

## Specifications table

**Table:** 

Subject area	*Computer Science*
More specific subject area	*Video processing; speech and facial recognition*
Type of data	*Digital video files*
How data was acquired	*Video recordings using a Sony 4K Handycam AX100*
Data format	*Raw*
Experimental factors	*N/A*
Experimental features	*Videos of the subjects’ head and upper shoulders while speaking. The dataset includes two videos per subject with all subjects speaking the same script. One is a longer script consisting of multiple sentences and the second is a single sentence.*
Data source location	*Fargo, North Dakota, USA*
Data accessibility	*Mendeley Data*
Related research article	

## Value of the data

• Data was collected in a controlled environment facilitating analysis and minimizing confounding factors.
• Includes multiple individuals with similar and dissimilar characteristics facilitating system testing and analysis of complex algorithms.
• Two videos are included, per subject, for use as training and validation sets.
• Can be used for training and testing of speech recognition and lip-motion recognition.

## 1 Data

Human speech recognition is used for numerous applications including transcription [1] and commanding computer workstations [2]. In support of these applications, techniques have been developed to overcome key technical challenges such as assessing recognition system performance confidence [3] and recognizing speech, despite background noise [4]. However, advances in speech synthesis have resulted in an inability to tell human speech from automated speech, in some applications [5]. Identifying that a human is speaking [6] can be a form of automated attack prevention. Identifying a particular human who is speaking could be used for authentication or as a factor in a multi-factor authentication system.

An alternate approach to recognizing humans and speech is by analyzing the human face. Lip reading has been performed by both software systems [7] and humans [8], with automated systems outperforming humans in some instances [8]. Facial recognition can also be used to identify humans [9], directly; however, other techniques [6] may be needed to verify that the is live, and not pre-recorded, input designed to fool a system. Facial imagery analysis has been used to aid speech recognition work [10] for synchronized audio and video feeds and, in particular, facilitate speech recognition in high-noise environments [11]. The analysis of facial imagery has also been used to assess other characteristics such as emotion and stress levels [12]. Problematically, creating synchronized audio and video lip movements has also been a study of work for some time [13] and efforts in this area may undermine the ability to validate subject liveness via combined audio and video analysis.

Sengupta et al. [14] proposed a technique for lip reading as part of authentication and others [15], [16], [17] have performed related speaker recognition and authentication work. Work is also ongoing in the audio speech recognition and lip reading domains, independent from security applications.

In support of the development and analysis of the aforementioned and other speech recognition algorithms, a variety of data sets have been collected. These have included data sets collected from pre-existing video footage [18] as well as specialty data set collected in application area environments, such as automobiles [19].

This dataset provides videos of 20 male subjects, with ages ranging from 18 to 26, which can be used for speech recognition, lip reading and combined speech and lip reading applications. Two videos were collected per subject. In the first, the subject read the FitnessGram Pacer Test script and in the second they said an arbitrary sentence. The data was collected in a controlled and well-lit environment with minimal environmental noise. This data was originally collected to train and test a compound biometric system that consists of lip-motion and voice authentication. The training videos averaged between 30 and 45 s and the testing videos averaged between 5 and 8 s each.

## 2 Experimental design, materials, and methods

This section discusses the procedures and equipment that was used to collect the video data. First, the equipment and configuration of the collection environment are discussed. Then, the data collection procedures are discussed.

### 2.1 Equipment and configuration

A 4K Sony Handycam AX100 was used to record the videos. A single Yongnuo YN600L light, set at 40% brightness, was used to illuminate the subject’s face. A standard projector screen was used as the background for all videos. Two Neewer LED500LRC lights were used as background lighting to eliminate any shadows that may be cast onto the projector screen by the lighting used to illuminate the subject’s face.

**Fig. 1:**
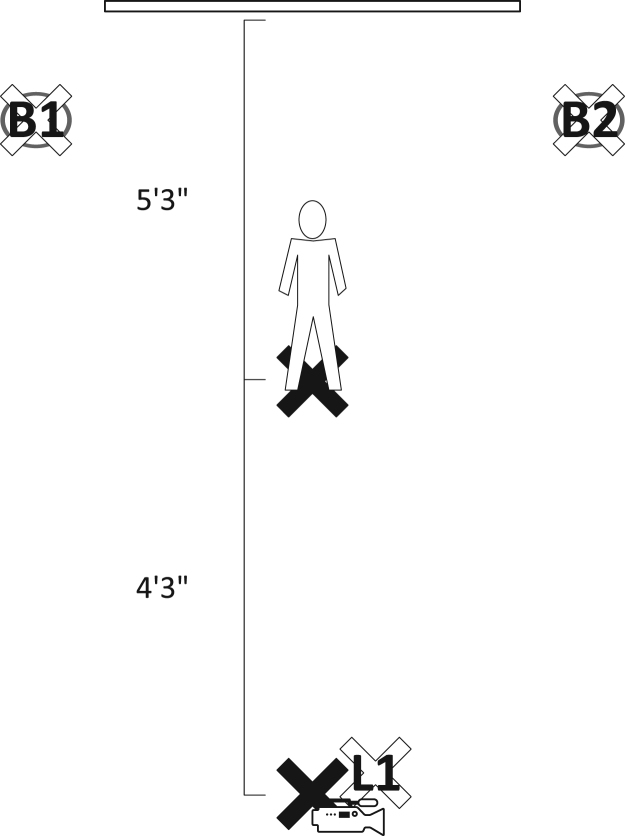
Diagram of subject, camera and lighting position.

**Fig. 2:**
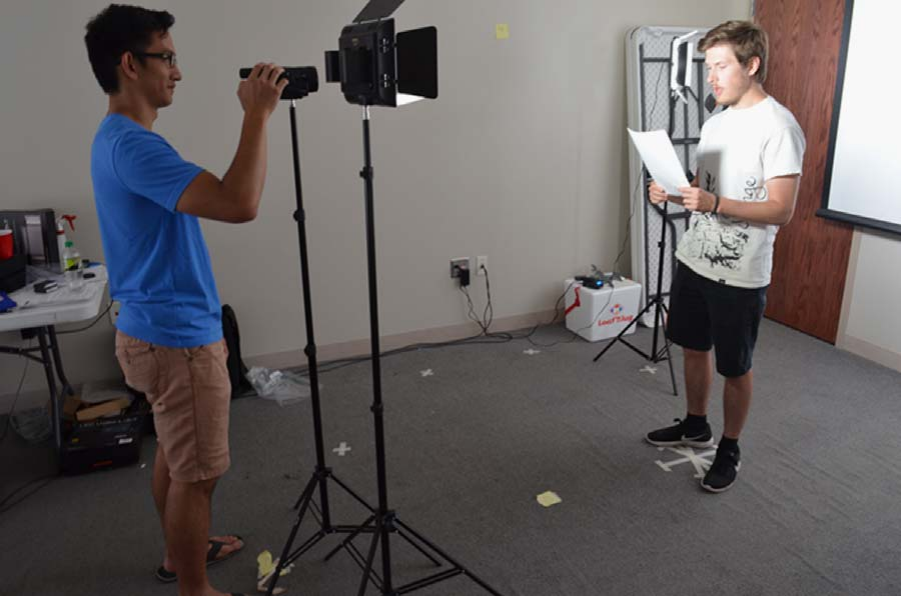
A subject is recorded while reading from the script.

The Handycam AX100 was placed on a tripod that was positioned approximately four feet and three inches from the subject, directly in front of the subject with the subject facing the camcorder and the projector screen behind the subject. The light for facial illumination was placed to the right of the camera. The locations of the subject and pieces of equipment are depicted in Fig. 1. Fig. 2 shows a subject being recorded. Both the camera and facial illumination light were positioned at the height of the middle of the subject’s face. The subject was located approximately five feet and three inches in front of the projector screen.

The Neewer LED500LRC background lights were positioned four feet and six inches to the left and three feet to the right of the subject, from the perspective of the camcorder. The projector screen was six feet tall and was elevated two feet and eight inches from the ground.

### 2.2 Data collection procedures

Twenty male subjects ranging in age between 18 and 26 were selected to participate. Each subject was given a copy of the script and instructed to stand on the center marker on the carpet, facing the camcorder. They were instructed to watch the camcorder and when they see that the red recording light is lit, to begin reciting the training script while keeping their head level and mouth visible to the camera.

**Fig. 3:**
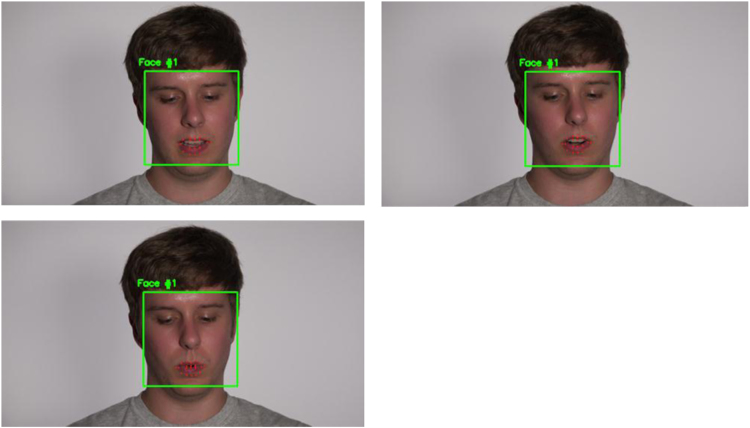
Depicts three frames from the video, showing how they can be processed to detect the subject’s face and lip positions and movement.

After finishing the training script recording, a second video was recorded of the subject reading the testing script, with the subject again instructed to start reading when they see that the red recording light is lit. Each video was manually reviewed to ensure that there was not disruptive background or extraneous noise or other interference. Three frames from the recording are shown in Fig. 3, which also depicts how the subject’s face and lips can be identified from the video.
