# Supplementary material for: Correlated lip motion and voice audio data
Source: Data Brief. 2018 Oct 18;21:856–60. doi: 10.1016/j.dib.2018.10.043 (PMC6218630; doi:10.1016/j.dib.2018.10.043)
Supplement: Supplementary file 1 — Supplementary material. [file mmc1.docx]

**Conflict of interest**

The authors declare no conflict of interest here.
